# Comparative Characterization of Stroma Cells and Ductal Epithelium in Chronic Pancreatitis and Pancreatic Ductal Adenocarcinoma

**DOI:** 10.1371/journal.pone.0094357

**Published:** 2014-05-05

**Authors:** Ole Helm, Ruben Mennrich, Domantas Petrick, Lisa Goebel, Sandra Freitag-Wolf, Christian Röder, Holger Kalthoff, Christoph Röcken, Bence Sipos, Dieter Kabelitz, Heiner Schäfer, Hans-Heinrich Oberg, Daniela Wesch, Susanne Sebens

**Affiliations:** 1 Institute for Experimental Medicine, Group Inflammatory Carcinogenesis, UKSH Campus Kiel, Kiel, Germany; 2 Institute of Immunology, UKSH Campus Kiel, Kiel, Germany; 3 Institute of Medical Informatics and Statistics, UKSH Campus Kiel, Kiel, Germany; 4 Institute of Experimental Cancer Research, UKSH Campus Kiel, Kiel, Germany; 5 Institute of Pathology, UKSH Campus Kiel, Kiel, Germany; 6 Institute of Pathology; University Tübingen, Tübingen, Germany; 7 Department of Internal Medicine I, Laboratory of Molecular Gastroenterology & Hepatology, UKSH Campus Kiel, Kiel, Germany; University of Szeged, Hungary

## Abstract

Pancreatic ductal adenocarcinoma (PDAC) is characterized by an extensive stroma being also present in chronic pancreatitis (CP). Using immunohistochemistry, the stroma of CP and PDAC was comprehensively analyzed and correlated with epithelial/carcinoma-related alterations and clinicopathological patient characteristics. While there were no significant differences between CP and PDAC regarding the distribution of CD3+ T cells and α-SMA+ fibroblasts, proportions of CD4+ and CD8+ T cells were significantly lower and numbers of CD25+(CD4+) and FoxP3+(CD4+) regulatory T cells were greater in PDAC compared with CP. Macrophages were more prevalent in CP, but localized more closely to carcinoma cells in PDAC, as were γδ-T cells. Duct-related FoxP3 and L1CAM expression increased from CP to PDAC, while vimentin expression was similarly abundant in both diseases. Moreover, stromal and epithelial compartments of well-differentiated tumors and CPs shared considerable similarities, while moderately and poorly differentiated tumors significantly differed from CP tissues. Analysis of 27 parameters within each pancreatic disease revealed a significant correlation of i) CD4+ and FoxP3+CD4+ T cells with FoxP3 expression in PDAC cells, ii) α-SMA+ fibroblasts with L1CAM expression and proliferation in PDAC cells, iii) CD3 and CD8 expression with γδ-TCR expression in both pancreatic diseases and iv) CD68+ and CD163+ macrophages with vimentin expression in PDAC cells. High expression of FoxP3, vimentin and L1CAM in PDAC cells as well as a tumor-related localization of macrophages each tended to correlate with higher tumor grade. Multivariate survival analysis revealed a younger age at time of surgery as a positive prognostic marker for PDAC patients with the most frequently operated disease stage T3N1M0. Overall this study identified several interrelationships between stroma and epithelial/carcinoma cells in PDACs but also in CP, which in light of previous experimental data strongly support the view that the inflammatory stroma contributes to malignancy-associated alterations already in precursor cells during CP.

## Introduction

Pancreatic ductal adenocarcinoma (PDAC) is the 4^th^ most lethal tumor disease with an overall 5-year-survival rate of ∼2% [1]. It is commonly diagnosed in an advanced stage, limiting curative therapeutic options to <20% of the patients. In addition, most of the PDAC patients do not respond to radio- or chemotherapy further worsening patient prognosis [2]. Thus, improving the diagnosis at an early disease stage as well as therapeutic options are both still urgently needed. For both, the identification of reliable biomarkers is of pivotal importance allowing the discrimination of PDAC from other benign pancreatic diseases on the one hand and prediction/improvement of therapeutic responses on the other hand.

Different precursor lesions have been identified which can give rise to PDAC. Besides intraductal papillary mucinous neoplasias (IPMN), mucinous cystic neoplasias (MCN) and atypical flat lesions (AFL), pancreatic intraepithelial neoplasias (PanIN) are the most frequent and best characterized precursor lesions of PDAC [3]. PanINs exhibit a ductal phenotype underscoring the view that PDAC originates from the ductal epithelium. Since PDAC is characterized by an extensive desmoplastic reaction accounting for up to 80% of the whole tumor mass, the tumor microenvironment has been regarded as a promising target to improve diagnosis and therapy of PDAC. The PDAC stroma is composed of extracellular matrix, fibroblasts, myofibroblasts and diverse immune cells [4], [5]. Interestingly, chronic pancreatitis (CP), which is regarded as high risk factor for the development of PDAC, also exhibits an extensive stromal response [4], [5]. Previous reports have demonstrated that the tumor-specific, non-neoplastic stromal cell population is highly variable and creates an immunosuppressive and tumor-promoting environment for the tumor cells of PDAC [4], [5]. High numbers of myofibroblasts (determined by α-SMA), M2-macrophages (determined by CD163 or CD204), regulatory T cells (T-regs determined by FoxP3 or CD25) and Th2 cells (determined by GATA-3^+^) have been generally found to correlate with tumor progression, reduced patient survival and worse prognosis [6]–[13]. Moreover, a recent study revealed that a stromal composition of CD4+ T cells^high^/CD8+ T cells^high^/T-reg^low^ and M1-macrophages^high^/M2-macrophages^low^ correlates with longer survival [13]. Beside the antigen-restricted T cell populations, γδ-T cells represent a promising T cell population in cancer therapy because of their ability of potently killing tumor cells in an non-HLA-restricted manner [14], [15]. However, little is known about their presence and role during PDAC development.

Upregulation of the adhesion molecule L1CAM (CD171) is associated with epithelial-mesenchymal-transition (EMT) which is also characterized by the upregulation of mesenchymal proteins such as vimentin [16]–[18]. L1CAM expression increases during PDAC progression in the ductal epithelium [16], [19], [20] and correlates with poor prognosis of PDAC patients [21]. Underscoring its protumorigenic function, L1CAM induces tumorigenicity of human pancreatic ductal epithelial (HPDE) cells, migration, apoptosis resistance and metastasis of HPDE and PDAC cells *in vitro* and *in vivo* [16], [19], [22] and is involved in the enrichment of T-regs in PDAC tissues (unpublished observations).

Besides its expression in T-regs and transiently in activated CD4+ T helper cells, the forkhead transcription factor 3 (FoxP3) has been also detected in various tumor cells, e.g. colorectal cancer and PDAC [23]–[25]. In PDAC, tumor related FoxP3 expression was associated with an immunosuppressive function *in vitro* [23] but an analysis regarding its impact on PDAC progression is still pending. In colorectal cancer, a high tumoral FoxP3 expression was already associated with poor prognosis [24].

Based on our profound experimental data pointing to an association between tumor-related L1CAM and FoxP3 and pancreatic stromal cells, this study compared immunohistochemically the expression of L1CAM and FoxP3 in pancreatic ductal epithelium (focusing on PanINs as the most frequent precursor lesion) and the stromal composition in CP and PDAC tissues. Moreover, to get further insights into the complex epithelia/carcinoma-stroma-interplay, the interrelationship of 27 parameters was determined. Finally, the predictive impact of these parameters was analyzed in a collective of PDAC patients with the most frequently operated disease stage T3N1M0.

## Materials and Methods

### Ethics statement

The research was approved by the ethics committee of the University Hospital Schleswig-Holstein as well as of the University Hospital Tübingen (reference number: D430/09, and 470/210BO1). Written informed consent was obtained from all patients.

### Patients & tissues

Pancreatic tissues were obtained from patients during surgery. Conservation of PDAC tissues and histopathological diagnosis were performed at the Institute of Pathology, UKSH Campus Kiel while conservation of CP tissues and their diagnostic were carried out at the Institute of Pathology, University Tübingen. In order to determine prognostic variables for the most frequent tumor stage, only PDAC patients with a tumor disease pathologically staged T3N1M0 were included in the study. Patient characteristics are summarized in **Table 1**. Besides the defined disease stage of T3N1M0, selection of tissue blocks was exclusively based on the presence of tumor cells in PDAC tissues. Selection of CP tissues was based on the presence of PanIN lesions.

**Table 1:** Characteristics of CP and PDAC patients.

Group	Parameter	Number of cases
**CP**	Patients	15
	Median age (range)	52 (40–69)
	Sex (male/female)	13/2
**PDAC**	Patients	42
	Median age (range)	65 (46–85)
	Sex (male/female)	24/18
	Tumor stage T3	42
	Nodal stage N1	42
	Metastasis stage M0	42
	Tumor grade 1: Well differentiated (G1)	6
	Tumor grade 2: Moderately differentiated (G2)	23
	Tumor grade 3: Poorly differentiated (G3)	13

### Immunohistochemistry

For immunohistochemistry, formalin fixed and paraffin embedded sections were used. Staining for CD3 (clone SP7, Fisher/Thermo Scientific, Schwerte, Germany), CD4 (clone 4B12, Leica Novocastra, Wetzlar, Germany), CD8 (clone C8/144B, Dako Cytomation, Hamburg, Germany), CD25 (clone 4C9, Leica Novocastra), CD163 (clone 10D6, Leica Novocastra), Ki67 (clone SP6, Fisher/Thermo Scientific), vimentin (clone Vim3B4, Dako Cytomation) and alpha-smooth muscle actin (α-SMA) (clone 1A4, Fisher/Thermo Scientific) was performed at the Institute of Pathology using an automated routine procedure. Staining of L1CAM was performed as previously described [19], [20], [26]. For staining of FoxP3 (clone hFoxy, ebioscience, Frankfurt a.M., Germany), TCR γδ (clone γ3.20, Thermo Scientific, Dreieich, Germany) and HLA-DR (CR3/43, Zytomed, Bargteheide, Germany) sections were further processed as previously described [19], [20]. Briefly, primary antibodies were used at the following previously titrated concentrations (FoxP3: 20 µg/ml in PBS/0.3% Triton-X100; TCR γδ : 3 µg/ml; HLA-DR: 1.02 µg/ml) and incubated 30 min at room temperature (RT). Detection of primary antibodies was performed by using EnVision-HRP (Dako, Hamburg, Germany) for 30 min at RT. Visualization was done by using the AEC Substrate (Dako) for 2–10 min. After final washing in PBS, sections were stained in Mayer's Haemalaun (AppliChem, Darmstadt, Germany) for 2 min. After washing in water for 10 min, cover slips were fixed with Kaiser's glycerine gelatine (Waldeck, Munster, Germany). For negative control, respective isotype control antibodies were used revealing no staining.

### Evaluation

Stained sections were evaluated twice in a blinded manner by scoring the staining intensity, reflecting the intensity of expression (given as intensity) and the extent of distribution (given as %-positivity of the whole section). In case of two discrepant results, sections were additionally evaluated by a second investigator. In CP tissues, all duct cell-related parameters are mainly based on the evaluation of PanINs, in PDAC tissues on the evaluation of tumor cells The stromal compartment in CP and PDAC tissues was defined as the whole pancreatic sample area excluding acinar cells, epithelial/tumor cells, lymphatic tissue and blood vessels. The scoring system for each variable is explained and listed in **Table 2** **,** **3** **,** **4**. Evaluation of the sections was carried out using an Axioplan 2.0 microscope (Zeiss, Jena, Germany). Pictures were taken using a Keyence BZ9000 microscope (Keyence, Neu-Isenburg, Germany).

**Table 2:** Scoring system used for pre-evaluation of the stromal and ductal epithelial/tumoral compartment in CP and PDAC tissues.

Score1	Score2	Score3	Score4	Score5
negative	<10% positive	10–50% positive	51–99% positive	100% positive

Parameters were evaluated using a 5-score system (**table 2**). The created groups were dicotomized by median and refitted into two groups (**table 3**) of <median (score 1) and ≥median (score 2). This determined different thresholds for medium, low and high expression markers. Evaluations of intensity, localization, numeric cell-counts and CD4∶CD8 ratio did not fit into the general scoring system and are described in **table 4**.

**Table 3:** Adapted scoring system for evaluation of the stromal and ductal epithelial/tumoral compartment in CP and PDAC tissues.

Scoring	Variable (related to)	Code
**Scoring of medium expression parameters** Score1: 0–10% positive, Score2: 11–100% positive	CD3+ (stroma)	**A**
	CD8+ (stroma)	**B**
	CD4+ (stroma)	**C**
	CD68+ (stroma)	**H1**
	CD163+ (stroma)	**I1**
	CD25+ (CD4+)	**E**
	FoxP3+ (CD4+)	**F**
	Ki67+ (% epithelium)	**N**
	FoxP3+ (% epithelium)	**O1**
	L1CAM+ (% epithelium)	**P1**
	Vimentin+ (% epithelium)	**R**
**Scoring of low expression parameters** Score1: negative, Score2: ≥1% positive	γδ+ (stroma)	**G1**
	γδ+ (ductal/tumor cells)	**G2**
	HLA-DR+ (stroma)	**K1**
**Scoring of high expression parameters** Score1: not 100% positive, Score2: 100% positive	α-SMA+ (stroma)	**L1**
	α-SMA^high^+ (α-SMA+)	**L2**
	Duct-related CD163+ (CD68+)	**I3**

**Table 4:** Scoring system for evaluation of special paramaters of the stromal and ductal epithelial/tumoral compartment in CP and PDAC tissues.

Scoring	Variable	Code
**Determination of expression intensity** Score1: none – low intensity, Score2: medium - strong intensity	FoxP3+ (Intensity epithelium)	**O2**
	L1CAM+ (Intensity epithelium)	**P2**
**Scoring of combined distribution and intensity** Score1: Score 1 in distribution and intensity, Score2: any higher scores in distribution and/or intensity	FoxP3 Score (%+Intensity epithelium)	**O3**
	L1CAM Score (%+Intensity epithelium)	**P3**
**Scoring of numeric cell counts in high magnification** Score1 = number <median, Score2 = number ≥median	Duct-related CD68+	**H3**
	Duct-related HLA-DR+	**K3**
**Determination of localization** Score1: close to epithelium, Score2: stromal, Score3: equally distributed, Score4 = not evaluable	CD68+ localization	**H2**
	CD163+ localization	**I2**
	HLA-DR+ localization	**K2**
**Determination of CD4:CD8 ratio** Score1: CD4 = CD8, Score2: CD4>CD8, Score3: CD4<CD8	CD4∶CD8 ratio	**D**

### Statistical analysis

All results were categorized and expressed as absolute and relative frequencies which were compared between groups by chi-square test. Association between stromal and ductal epithelium related parameters within pancreatic tissues of CP patients and PDAC patients were analyzed and tested by chi-square test. Survival curves were estimated according to Kaplan-Meier method and possible influence factors were identified by log-rank test. All tests were at a level of significance of 5% without adjusting for multiple testing. Statistical analysis was performed using SPSS 17.0.

## Results

### Comparison of stromal and ductal epithelial/tumoral compartment in CP and PDAC

**Figure 1:**
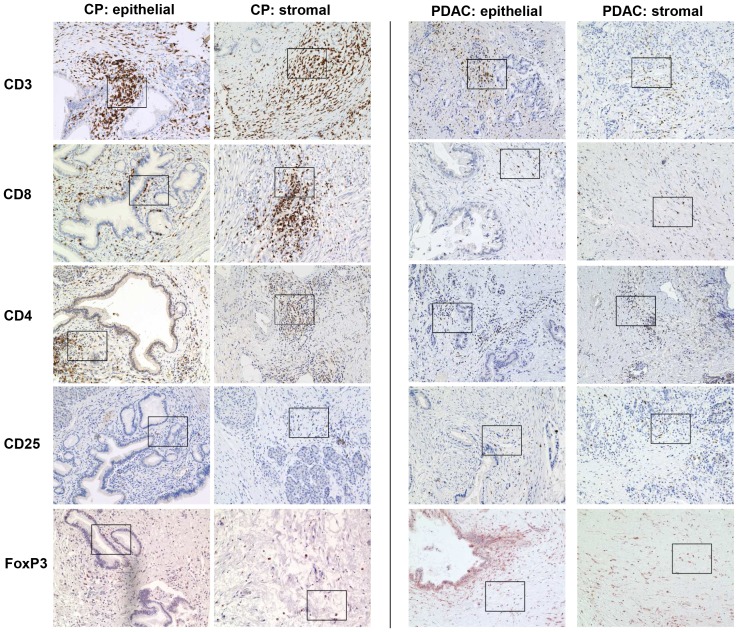
Immunhistochemical characterization of the stromal compartment in CP and PDAC tissues. Representative stainings present the distribution of CD3, CD8, CD4, CD25 and FoxP3 in stromal cells located in the pancreatic stroma distant (stromal) or in close proximity to ductal epithelial/carcinoma cells (epithelial) in CP and PDAC tissues. Magnification ×200.

First we examined the cellular composition of the stromal and ductal epithelial/tumoral compartment in 15 CP and 42 PDAC using the markers listed in **Table 5**. In the stromal compartment CD3+, CD4+ and CD8+ T cells were significantly more abundant in CP compared with PDAC (**Table 5** **,** **Figure 1** **+** **2**). Although CPs exhibited higher numbers of CD4+ T cells, the proportion of CD25+ and Foxp3+ cells in this T cell population indicative for regulatory T cells (T-regs) was markedly higher in PDAC than in CPs (58% with score 2 versus 20–26% with score 2, respectively). In contrast, stromal γδ-T cells were detected at higher levels (score 2) in 80% of CPs but only in 50% of PDACs. Moreover, γδ-T cells within or adjacent to the ductal/tumoral epithelium were localized in 67% of the CP as well as in 44% of the PDAC patients (**Figure 3** **+** **4**). In addition, γδ-T cells accumulated more intensively within the ductal/tumoral epithelium in PDAC patients compared to CP patients (**Figure 3** **+** **4**). Similar to the findings on γδ-T cells, CD68-positive macrophages were found at higher numbers (score 2) in 80% of CPs but only in 50% of PDACs (**Figure 3** **+** **4**). Interestingly, the distribution of macrophages significantly differed: while macrophages were generally equally distributed in the stroma and close to pancreatic ducts in CP, CD68+ macrophages were predominantly located adjacent to the tumor cells in over 50% of PDACs. A similar observation was made for CD163, a marker for M2-macrophages. HLA-DR, a marker for M1-macrophages, was also more abundant in CPs (86%) compared with PDACs (59%). Since HLA-DR was found in areas with high numbers of CD163+ macrophages leads to the conjecture that macrophages might share M1- and M2-characteristics simultaneously. However, in PDACs, HLA-DR+ macrophages were equally distributed throughout the tumor and not enriched in vicinity of the tumor cells (**Figure 3** **+** **4**). In contrast to T cells and macrophages, fibroblasts and predominantly myofibroblasts (α-SMA^high^+ cells) tended to accumulate at higher numbers in PDACs compared to CPs (61% versus 40% with score 2 for fibroblasts and 42% versus 20% with score 2 for myofibroblasts, respectively) (**Figure 3** **+** **4**).

**Figure 2:**
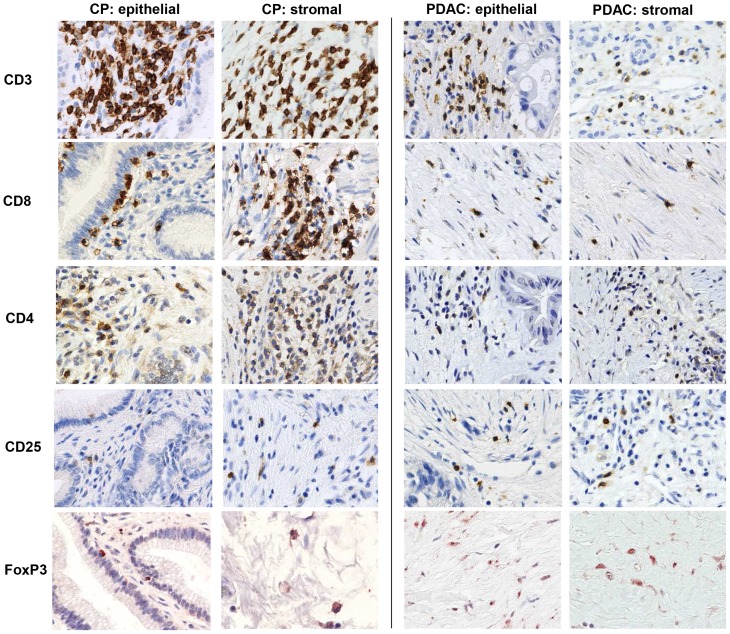
Immunhistochemical characterization of the stromal compartment in CP and PDAC tissues. Representative stainings present the distribution of CD3, CD8, CD4, CD25 and FoxP3 in stromal cells located in the pancreatic stroma distant (stromal) or in close proximity to ductal epithelial/carcinoma cells (epithelial) in CP and PDAC tissues. Magnification ×800.

**Figure 3:**
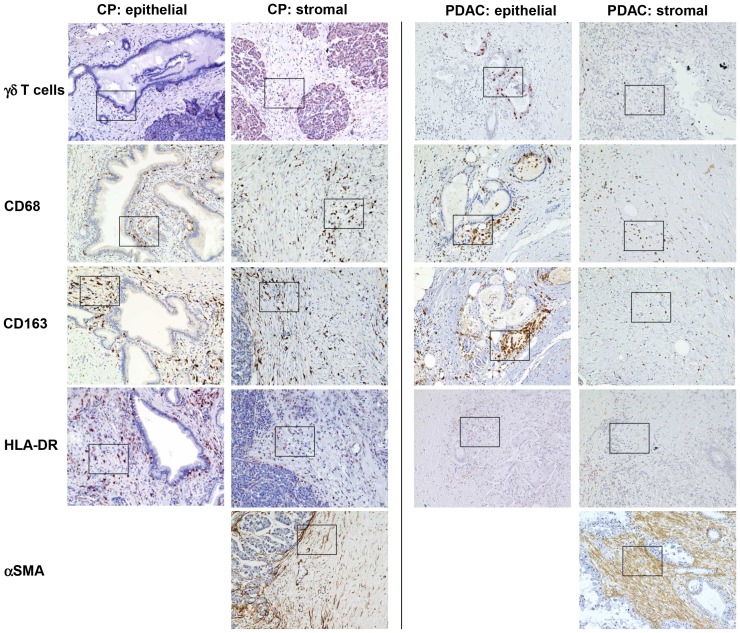
Immunhistochemical characterization of the stromal compartment in CP and PDAC tissues. Representative stainings present the distribution of γδ-TCR, CD68, CD163, HLA-DR and α-SMA in stromal cells located in the pancreatic stroma distant (stromal) or in close proximity to ductal epithelial/carcinoma cells (epithelial) in CP and PDAC tissues. Magnification ×200.

**Figure 4:**
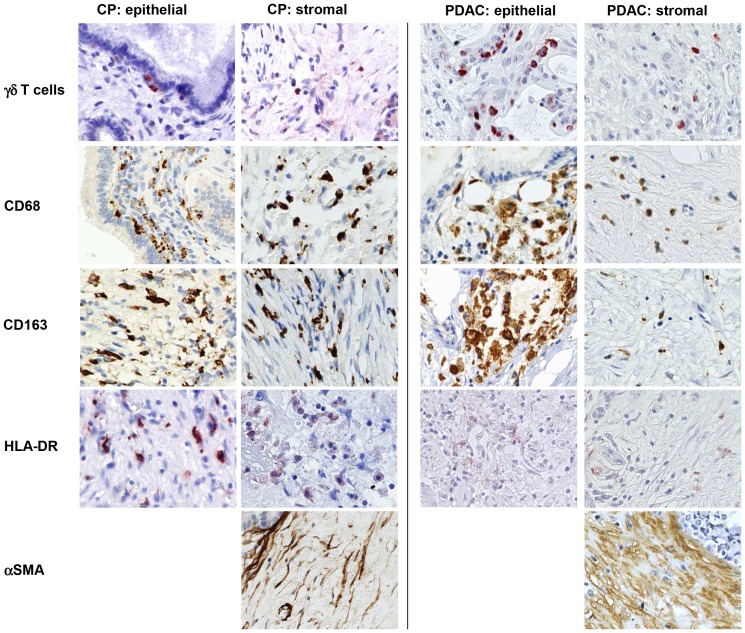
Immunhistochemical characterization of the stromal compartment in CP and PDAC tissues. Representative stainings present the distribution of γδ-TCR, CD68, CD163, HLA-DR and α-SMA in stromal cells located in the pancreatic stroma distant (stromal) or in close proximity to ductal epithelial/carcinoma cells (epithelial) in CP and PDAC tissues. Magnification ×800.

**Table 5:** Interrelationship between stromal and ductal/tumor cell-related parameters in pancreatic tissues of CP and PDAC patients.

Variable	Code	CP (n = 15)					PDAC (n = 42)					p-value
Stromal compartment (related to)		Number of cases (Score)					Number of cases (Score)					
CD3+ (stroma)	A	0 (1)	15 (2)				5 (1)	37 (2)				**0.311**
CD8+ (stroma)	B	1 (1)	13 (2)			1 (NE)	13 (1)	29 (2)				0.150
CD4+ (stroma)	C	1 (1)	14 (2)				21 (1)	21 (2)				**<0.01**
CD25+ (CD4+)	E	12 (1)	3 (2)				17 (1)	24 (2)			1 (NE)	**0.015**
FoxP3+ (CD4+)	F	11 (1)	4 (2)				17 (1)	24 (2)			1 (NE)	0.068
γδ+ (stroma)	G1	3 (1)	12 (2)				20 (1)	21 (2)			1 (NE)	0.069
γδ+ (ductal/tumor cells)	G2	5 (1)	10 (2)				23 (1)	18 (2)			1 (NE)	0.227
CD68+ (stroma)	H1	4 (1)	11 (2)				21 (1)	19 (2)			2 (NE)	0.129
CD68+ localization	H2	0 (E/T)	7 (S)	8 (E)			22 (E/T)	6 (S)	13 (E)		1 (NE)	**<0.01**
CD163+ (stroma)	I1	4 (1)	11 (2)				21 (1)	20 (2)			1 (NE)	0.134
CD163+ localization	I2	0 (E/T)	7 (S)	8 (E)			22 (E/T)	6 (S)	13 (E)		1 (NE)	**<0.01**
HLA-DR+ (stroma)	K1	2 (1)	13 (2)				15 (1)	25 (2)			2 (NE)	0.109
HLA-DR+ localization	K2	2 (E/T)	4 (S)	7 (E)	2 (ND)		9 (E/T)	5 (S)	11 (E)	15 (ND)	2 (NE)	0.165
α-SMA+ (stroma)	L1	10 (1)	3 (2)			2 (NE)	23 (1)	18 (2)			1 (NE)	0.211
α-SMA ^high^+ (α-SMA+)	L2	7 (1)	6 (2)			2 (NE)	15 (1)	26 (2)			1 (NE)	0.338
**Ductal/tumoral compartment**												
Ki67+ (%)	N	11 (1)	1 (2)			3 (NE)	9 (1)	30 (2)			3 (NE)	**<0.01**
FoxP3+ (%)	O1	11 (1)	4 (2)				17 (1)	24 (2)			1 (NE)	0.068
FoxP3+ (intensity)	O2	10 (1)	5 (2)				7 (1)	34 (2)			1 (NE)	**<0.01**
L1CAM+ (%)	P1	5 (1)	10 (2)				19 (1)	22 (2)			1 (NE)	0.543
L1CAM+ (intensity)	P2	13 (1)	2 (2)				20 (1)	21 (2)			1 (NE)	**0.014**
Vimentin+ (%)	R	7 (1)	8 (2)				23 (1)	18 (2)			1 (NE)	0.746

**ND** = not detectable; **NE** = not evaluable; **E/T** = located close to **E**pithelial/**T**umor cells; **S** = located in **S**troma; **E** = equally distributed close to epithelial/tumor cells and in stroma.

**Figure 5:**
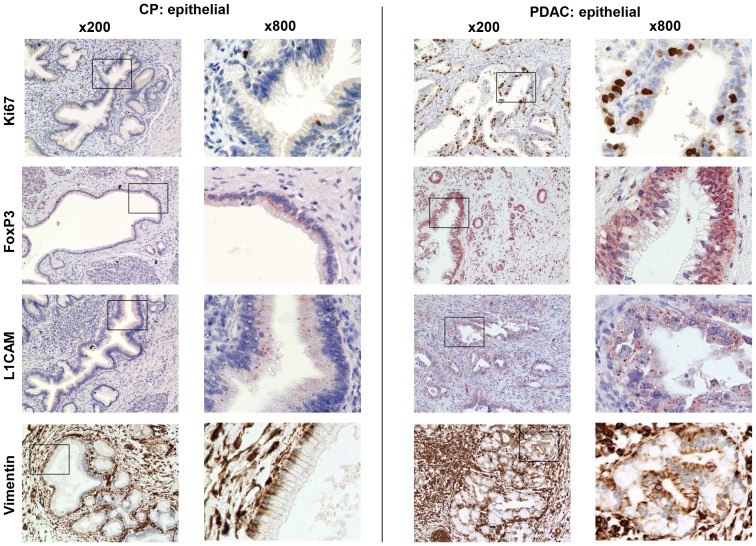
Immunhistochemical characterization of the ductal epithelium in CP and PDAC tissues. Representative stainings represent the distribution of Ki67, FoxP3, L1CAM and vimentin in ductal epithelial/carcinoma cells (epithelial) in CP and PDAC tissues. Magnification ×200 and ×800.

Comparing ductal epithelial cells in CP with carcinoma cells in PDAC (**Table 5** **,** **Figure 5**) showed that carcinoma cells exhibited a significantly increased proliferative activity indicated by a higher percentage of Ki67+ carcinoma cells compared to ductal epithelial cells in CPs. Moreover, FoxP3 expression in ductal epithelium was already present in 80% of CPs albeit in only few cells (% cells score 1) and with a low expression (intensity score 1). In contrast, FoxP3 expression was markedly increased in carcinoma cells being detectable at score 2 in 60% (% cells) and 80% (intensity), respectively. Immunostaining of EMT-associated L1CAM revealed that >60% of the pancreatic duct epithelia in CPs expressed L1CAM (% cells score 2) while in PDACs, approximately 50% of the tumor cells were characterized by a similar expression score. However, the intensity of L1CAM expression was markedly greater in PDACs (50% with score 2) than in CP tissues (13% with score 2). Vimentin expression as another indicator for EMT induction in ductal epithelial cells was already detectable in 47% of CP tissues and similarly expressed in carcinoma cells in PDAC tissues.

Collectively, our findings show marked differences between the stroma of CP and PDAC. Thus, the desmoplastic stroma of PDAC harbors a lower number of CD8+ and γδ-T cells but higher numbers of T-regs compared with CP. These findings lend support to the hypothesis that the stromal compartment undergoes fundamental changes during progression to an invasive PDAC being characterized by the acquisition of immunosuppressive properties. Interestingly, duct-related expression of FoxP3 and L1CAM, both found already in CP, was significantly elevated in PDACs. This observation together with the fact that vimentin expression was already abundant in the ductal epithelium of PanINs in CP, too, suggests that malignancy-associated alterations might also start during CP.

### Comparison of stromal and ductal epithelial/tumoral compartment in CP and PDAC considering tumor grading

In order to clarify whether dedifferentiation of tumor cells is associated with alterations in the stromal compartment, the stromal and epithelial compartment in CP tissues were compared with those in PDAC tissues considering tumor grading. The detailed results from each single comparison are presented in **Tables S1, S2, S3**. As summarized in **Table 6**, well differentiated (G1) tumors significantly differed from CP tissues only in the number of CD4+ T cells (code C) that were already less abundant in PDAC tissues and L1CAM expression (P2) being already elevated in PDAC cells compared to the ductal epithelium in CP tissues. In contrast, moderately (G2) and poorly (G3) differentiated tumors significantly differed with respect to more parameters from CP tissues. In detail, the number of CD8+ T cells (B) was significantly reduced in G3 tumors but not in G2 and G1 tumors, while the number of CD25+ (CD4+) T cells was already increased in G2 tumors. Differences regarding the amount and localization of macrophages (H1, H2, I1, I2) as well as proliferation (N) and FoxP3 expression (O1+O2) were also evident in G2 tumors. In contrast, no significant differences regarding the number of CD3+ T cells, γδ-T cells, α-SMA fibroblasts and vimentin expression could be determined. Overall these data suggest that not only the epithelial/tumoral but also the stromal compartment in well differentiated (G1) PDACs still share more similarities with those in CP tissues than those of G2 and G3 tumors having undergone dedifferentiation.

**Table 6:** Results from statistical comparison of stromal and ductal/tumor cell-related parameters in pancreatic tissues of CP patients and patients with well (G1), moderately (G2) and poorly differentiated (G3) PDAC.

Variable	Code	CP vs G1	CP vs G2	CP vs G3
**Stromal compartment (related to)**				
CD3+ (stroma)	A	np	0.264	0.206
CD8+ (stroma)	B	0.521	0.217	**0.033**
CD4+ (stroma)	C	**0.011**	**<0.01**	0.069
CD25+ (CD4+)	E	0.291	**0.041**	0.051
FoxP3+ (CD4+)	F	0.146	0.19	0.056
γδ+ (stroma)	G1	0.120	0.294	0.057
γδ+ (ductal/tumor cells)	G2	0.331	0.583	0.182
CD68+ (stroma)	H1	0.613	**0.045**	0.689
CD68+ localization	H2	0.157	**<0.01**	**0.015**
CD163+ (stroma)	I1	0.613	0.052	0.689
CD163+ localization	I2	0.157	**<0.01**	**0.015**
HLA-DR+ (stroma)	K1	1.000	0.273	**0.037**
HLA-DR+ localization	K2	0.955	0.336	0.068
α-SMA+ (stroma)	L1	1.000	0.071	1.000
α-SMA^high^+ (α-SMA+)	L2	0.268	0.468	0.411
**Ductal/tumoral compartment**				
Ki67+ (%)	N	0.191	**<0.01**	**<0.01**
FoxP3+ (%)	O1	0.354	**<0.01**	**<0.01**
FoxP3+ (intensity)	O2	0.331	**<0.01**	**<0.01**
L1CAM+ (%)	P1	1.000	0.738	0.266
L1CAM+ (intensity)	P2	**0.031**	**0.016**	0.198
Vimentin+ (%)	R	1.000	0.169	0.460

**np** = not possible (both parameters are constant).

### Interrelationship between stromal and ductal epithelium-related parameters in pancreatic tissues of CP patients

Next, we tested the hypothesis whether the expression of stromal markers in CP correlates with each other and with epithelial markers of non-neoplastic pancreatic duct epithelium and PanIN lesions, respectively (**Table 7**). The following most striking interrelationships could be noted mostly showing statistical significance. Codes for the respective variables used in the tables are shown in brackets. i) A low FoxP3 (O1+O3) and L1CAM (P1+P3) expression in the ductal epithelium was correlated with a predominance of CD4+ T cells over CD8+ T cells (D) and vice versa. iii) CD68+ and CD163+ macrophages correlated regarding their proportion (H1 and I1) as well as their localization within the tissue (H2, I2). The latter applied also for HLA-DR+ macrophages (K2). iv) HLA-DR+ macrophages showed no preferential localization in CP tissues (K1+K2). However, at higher numbers these cells predominantly localized close to the ductal epithelium (K1+K3). v) Low numbers of myofibroblasts (L2) were correlated with a low expression of FoxP3 (O1) and tended to correlate with L1CAM expression in pancreatic ducts (P1+P3). Vice versa, great numbers of myofibroblasts tended to correlate with high L1CAM expression in the ductal epithelium (P1+P3). vi) Finally, a high FoxP3 score (O3) was associated with a high L1CAM score (P3) in the ductal epithelium in CP tissues.

**Table 7:** Association between stromal and ductal epithelium-related parameters in pancreatic tissues of CP patients, expressed as p-values from chi-square test.

Variable	Code	A	B	C	D	E	F	H1	H2	H3	I1	I2	I3	K1	K2	K3	L1	L2	N	O1	O2	O3	P1	P2	P3	R
CD3+ (stroma)	**A**	X	np	np	np	np	np	np	np	np	np	np	np	np	np	np	np	np	np	np	np	np	np	np	np	np
CD8+ (stroma)	**B**		X	ns	ns	ns	ns	ns	ns	ns	ns	ns	np	ns	ns	ns	ns	ns	ns	ns	ns	ns	ns	ns	ns	ns
CD4+ (stroma)	**C**			X	ns	ns	ns	ns	ns	ns	ns	ns	np	ns	ns	ns	ns	ns	ns	ns	ns	ns	ns	ns	ns	ns
CD4∶CD8 ratio	**D**				X	ns	ns	ns	ns	ns	ns	ns	np	ns	ns	ns	ns	ns	ns	**0.055**	ns	**0.026**	**0.031**	ns	**0.031**	ns
CD25+ (CD4+)	**E**					X	ns	ns	ns	ns	ns	ns	np	ns	ns	ns	ns	ns	ns	ns	ns	ns	ns	ns	ns	ns
FoxP3+ (CD4+)	**F**						X	ns	ns	ns	ns	ns	np	ns	ns	ns	ns	ns	ns	ns	ns	ns	ns	ns	ns	ns
CD68+ (stroma)	**H1**							X	ns	ns	**0.001**	ns	np	ns	ns	ns	ns	ns	ns	ns	ns	ns	ns	ns	ns	ns
CD68+ localization	**H2**								X	ns	ns	**<0.01**	np	ns	**0.005**	ns	**0.07**	ns	ns	ns	ns	ns	ns	ns	ns	ns
Duct-related CD68+	**H3**									X	ns	ns	np	ns	ns	ns	ns	ns	ns	ns	ns	ns	ns	ns	ns	ns
CD163+ (stroma)	**I1**										X	ns	np	ns	ns	ns	ns	ns	ns	ns	ns	ns	ns	ns	ns	ns
CD163+ localization	**I2**											X	np	ns	**0.005**	ns	**0.07**	ns	ns	ns	ns	ns	ns	ns	ns	ns
Duct-related CD163+	**I3**												X	np	np	np	np	np	np	np	np	np	np	np	np	np
HLA-DR+ (stroma)	**K1**													X	**0.002**	**0.011**	ns	ns	ns	ns	ns	ns	ns	ns	ns	ns
HLA-DR+ localization	**K2**														X	**0.03**	ns	ns	ns	ns	ns	ns	ns	ns	**0.084**	ns
Duct-related HLA-DR+	**K3**															X	ns	ns	ns	ns	ns	ns	ns	ns	ns	ns
α-SMA+ (stroma)	**L1**																X	ns	ns	ns	ns	ns	ns	ns	ns	ns
α-SMA^high^+ (α-SMA+)	**L2**																	X	ns	**0.02**	ns	ns	**0.07**	ns	**0.07**	ns
Ki67+ (%)	**N**																		X	ns	ns	ns	ns	ns	ns	ns
FoxP3+ (%)	**O1**																			X	ns	**0.03**	ns	ns	ns	ns
FoxP3+ (intensity)	**O2**																				X	**<0.01**	ns	ns	ns	ns
FoxP3 Score (%+Int.)	**O3**																					X	**0.026**	ns	**0.026**	ns
L1CAM+ (%)	**P1**																						X	ns	**<0.01**	ns
L1CAM+ (intensity)	**P2**																							X	ns	ns
L1CAM Score (%+Int.)	**P3**																								X	ns
Vimentin+ (%)	**R**																									X

**np** = not possible (one parameter is a constant); **ns** = not significant.

### Interrelationship between stromal and carcinoma-related parameters in pancreatic tissues of PDAC patients

Next, we tested the hypothesis whether the expression of desmoplastic stroma markers in PDAC correlate with each other and with carcinoma cells (**Table 8**). The following most striking interrelationships could be noted mostly showing statistical significance. Codes for the respective variables used in the tables are shown in brackets. i) A high abundance of CD3+ T cells (A) was associated with high numbers of CD8+ (B) and CD4+ (C) T cells. A dominance of CD8+ T cells over CD4+ T cells (D) was associated with high numbers of FoxP3+ (CD4+) T cells (F) while low numbers of CD8+ T cells (B) were neither associated with high numbers of HLA-DR+ macrophages (K1) nor with their tumor-related localization (K3). In contrast, tissues with a high percentage of HLA-DR+ tumor-related macrophages (K3) were characterized by high numbers of CD25+ (CD4+) T cells (E). A similar trend, although not being significant, was observed for CD68+ macrophages located in close proximity to carcinoma cells (H3) as well as to FoxP3+(CD4+) T cells (F). ii) When CD4+ T cells (C) highly accumulated in the stroma, these cells showed a dominance over CD8+ T cells (D) and vice versa. In addition, high numbers of CD4+ T cells (C) correlated with a low score of CD25+ (CD4+) (E) and FoxP3+ (CD4+) (F) T cells and vice versa. iii) Low numbers of CD4+ (C) but high numbers of FoxP3+ (CD4+) T cells (F) were associated with high FoxP3 expression in tumor cells (O1). iv) The percentage and intensity of FoxP3 (O1–O3) positively correlated with L1CAM expression (P1+P2) in tumor cells. Moreover, a high percentage of FoxP3+ tumor cells (O1) tended to correlate with higher grading (S). v) The percentage (H1) and the localization (H2) of CD68+ macrophages positively correlated with both of CD163+ macrophages (I1, I2) and with the localization of HLA-DR+ macrophages (K2). A high number of CD68+ macrophages (H1) was associated with higher numbers of CD163+ macrophages (I1). At low numbers, CD68+ (H1) and CD163+ (I1) macrophage predominantly localized close to tumor cells while at higher numbers, both macrophage populations were equally distributed throughout the tissues, respectively (H2,I2). Moreover, when only low numbers of CD68+ macrophages were localized in close proximity to tumor cells (H3), these macrophages rarely expressed HLA-DR (K3). vi) A tumor-related localization of CD68+ (H2) and CD163+ (I2) macrophages was associated with a high abundance of myofibroblasts (L2). vii) In contrast, low numbers of fibroblasts (L1) were correlated with an increased percentage of Ki67+ carcinoma cells (N) as well as a high accumulation of CD3+ (A) and CD4+ (C) T cells. No correlation with the presence of CD8+ (B), CD25+ (CD4+) (E) or FoxP3+ (CD4+) (F) was found. Accordingly, a dominance of CD8+ over CD4+ T cells (D) was detected by trend in the presence of high fibroblast numbers (L1). viii) A high abundance of fibroblasts (L1) in the tumoral stroma correlated with increased L1CAM expression in the carcinoma cells (P2) and high numbers of CD68+ (H1) and CD163+ (I1) macrophages correlated with elevated vimentin expression in tumor cells (R). ix) Finally, localization of CD68+ (H2) and CD163+ (I2) macrophages close to carcinoma cells as well as high expression of FoxP3 (O2), L1CAM (P3) and vimentin (R), respectively, tended to be associated with higher grading (S).

**Table 8:** Association between stroma and tumor cell-related variables in pancreatic tissues of PDAC patients, expressed as p-values from chi-square test.

Variable	Code	A	B	C	D	E	F	H1	H2	H3	I1	I2	I3	K1	K2	K3	L1	L2	N	O1	O2	O3	P1	P2	P3	R	S
CD3+ (stroma)	**A**	X	**0.002**	**0,048**	**ns**	ns	ns	ns	ns	ns	ns	ns	np	ns	ns	ns	ns	ns	ns	ns	ns	ns	ns	ns	ns	ns	ns
CD8+ (stroma)	**B**		X	ns	ns	ns	ns	ns	ns	ns	ns	ns	np	**0.028**	ns	**0.003**	ns	ns	ns	ns	ns	ns	ns	ns	ns	ns	ns
CD4+ (stroma)	**C**			**X**	**<0.01**	**0.028**	**0.028**	ns	ns	ns	ns	ns	np	ns	ns	ns	**0.062**	ns	**0.078**	**0.004**	ns	ns	ns	ns	ns	ns	ns
CD4∶CD8 ratio	**D**				X	ns	**0.035**	ns	ns	ns	ns	ns	np	ns	ns	ns	**0,006**	ns	ns	ns	ns	ns	ns	ns	**0.031**	ns	ns
CD25+ (CD4+)	**E**					X	ns	ns	ns	ns	ns	ns	np	ns	ns	**0.012**	ns	ns	ns	ns	ns	ns	ns	ns	ns	ns	ns
FoxP3+ (CD4+)	**F**						X	ns	ns	ns	ns	ns	np	ns	ns	ns	ns	ns	**0.052**	**0.014**	ns	ns	ns	ns	ns	ns	ns
CD68+ (stroma)	**H1**							X	**0.083**	ns	**<0.01**	**0.083**	np	ns	ns	ns	ns	ns	ns	ns	ns	ns	ns	ns	ns	**0.017**	ns
CD68+ localization	**H2**								X	ns	ns	**0.000**	np	ns	**0.009**	ns	ns	**0.027**	ns	ns	ns	ns	ns	ns	ns	ns	**0.009**
Duct-related CD68+	**H3**									X	ns	ns	np	ns	**0.007**	**0.008**	ns	**0.007**	ns	ns	ns	ns	**0.010**	ns	**0.041**	ns	ns
CD163+ (stroma)	**I1**										X	ns	np	ns	ns	ns	ns	ns	ns	ns	ns	ns	ns	ns	ns	**0.026**	ns
CD163+ localization	**I2**											X	np	ns	**0.009**	ns	ns	**0.027**	ns	ns	ns	ns	ns	ns	ns	ns	**0.009**
Duct-related CD163+	**I3**												X	np	np	np	np	np	np	np	np	np	np	np	np	np	np
HLA-DR+ (stroma)	**K1**													X	**<0.01**	**<0.01**	ns	ns	ns	ns	ns	ns	ns	ns	ns	ns	ns
HLA-DR+ localization	**K2**														X	**<0.01**	ns	ns	ns	ns	ns	ns	ns	ns	ns	ns	ns
Duct-related HLA-DR+	**K3**															X	ns	ns	ns	ns	ns	ns	ns	ns	ns	ns	ns
α-SMA+ (stroma)	**L1**																X	ns	**0.026**	ns	ns	ns	ns	**0.031**	ns	ns	ns
α-SMA^high^+ (α-SMA+)	**L2**																	X	ns	ns	ns	ns	ns	ns	ns	ns	ns
Ki67+ (%)	**N**																		X		ns	ns	ns	ns	ns	ns	ns
FoxP3+ (%)	**O1**																			X	**0.082**	**0.001**	ns	ns	ns	ns	**0.061**
FoxP3+ (intensity)	**O2**																				X	**0.001**	ns	ns	ns	ns	ns
FoxP3 Score (%+Int.)	**O3**																					X	ns	ns	ns	ns	ns
L1CAM+ (%)	**P1**																						X	**0.001**	**<0.01**	ns	ns
L1CAM+ (intensity)	**P2**																							X	**<0.01**	ns	ns
L1CAM Score (%+Int.)	**P3**																								X	ns	**0.069**
Vimentin+ (%)	**R**																									X	**0.051**
Grading	**S**																										X

**np** = not possible (one parameter is a constant); **ns** = not significant.

### Interrelationship between stromal and ductal epithelium/carcinoma-related γδ-T cells in pancreatic tissues of CP and PDAC patients

Since the presence of γδ-T cells has been rarely investigated in CP and PDAC tissues at all, a more detailed analysis on these cells has been performed in this study (scoring system see **Table 9**). As shown in **Table 10**, γδ-T cells were located in the same area of the stroma and ductal/tumoral epithelium as CD3- and CD8-positive T cells in both pancreatic diseases. Furthermore, γδ-T cells accumulated more intensively in the malignant epithelium and the stroma close to the tumor cells in PDACs, whereas in CP tissues γδ-T cells were distributed throughout the entire stromal compartment (**Table 10** **,** **Figure 3** **+** **4**).

**Table 9:** Scoring system used for evaluation of γδ T cells in the stromal and ductal/tumoral compartment in CP and PDAC tissues.

Scoring	Variable (related to)	Code
Score1: negative, Score2: ≥1% positive	γδ+ (stroma)	**G1**
	CD3+ (stroma)	**G1A**
	CD8+ (stroma)	**G1B**
	γδ+ (ductal/tumor cells)	**G2**
	CD3+ (ductal/tumor cells)	**G2A**
	CD8+ (ductal/tumor cells)	**G2B**

**Table 10:** Association between stromal and ductal/tumor cell-related parameters in pancreatic tissues of CP and PDAC patients, expressed as p- values from chi-square test.

CP	Variable(related to)	Code	G1	G1A	G1B	G2	G2A	G2B
	γδ+ (stroma)	**G1**	X	<0.01	<0.01	0.02	0.02	0.02
	CD3+ (stroma)	**G1A**		X	<0.01	0.02	0.02	0.02
	CD8+ (stroma)	**G1B**			X	0.02	0.02	0.02
	γδ+ (ductal cells)	**G2**				X	<0.01	<0.01
	CD3+ (ductal cells)	**G2A**					X	<0.01
	CD8+ (ductal cells)	**G2B**						X

PDAC	Variable(related to)	Code	G1	G1A	G1B	G2	G2A	G2B
	γδ+ (stroma)	**G1**	X	<0.01	<0.01	<0.01	<0.01	<0.01
	CD3+ (stroma)	**G1A**		X	<0.01	<0.01	<0.01	<0.01
	CD8+ (stroma)	**G1B**			X	<0.01	<0.01	<0.01
	γδ+ (tumor cells)	**G2**				X	<0.01	<0.01
	CD3+ (tumor cells)	**G2A**					X	<0.01
	CD8+ (tumor cells)	**G2B**						X

### Correlation of stromal and carcinoma-related parameters with patient survival

**Figure 6:**
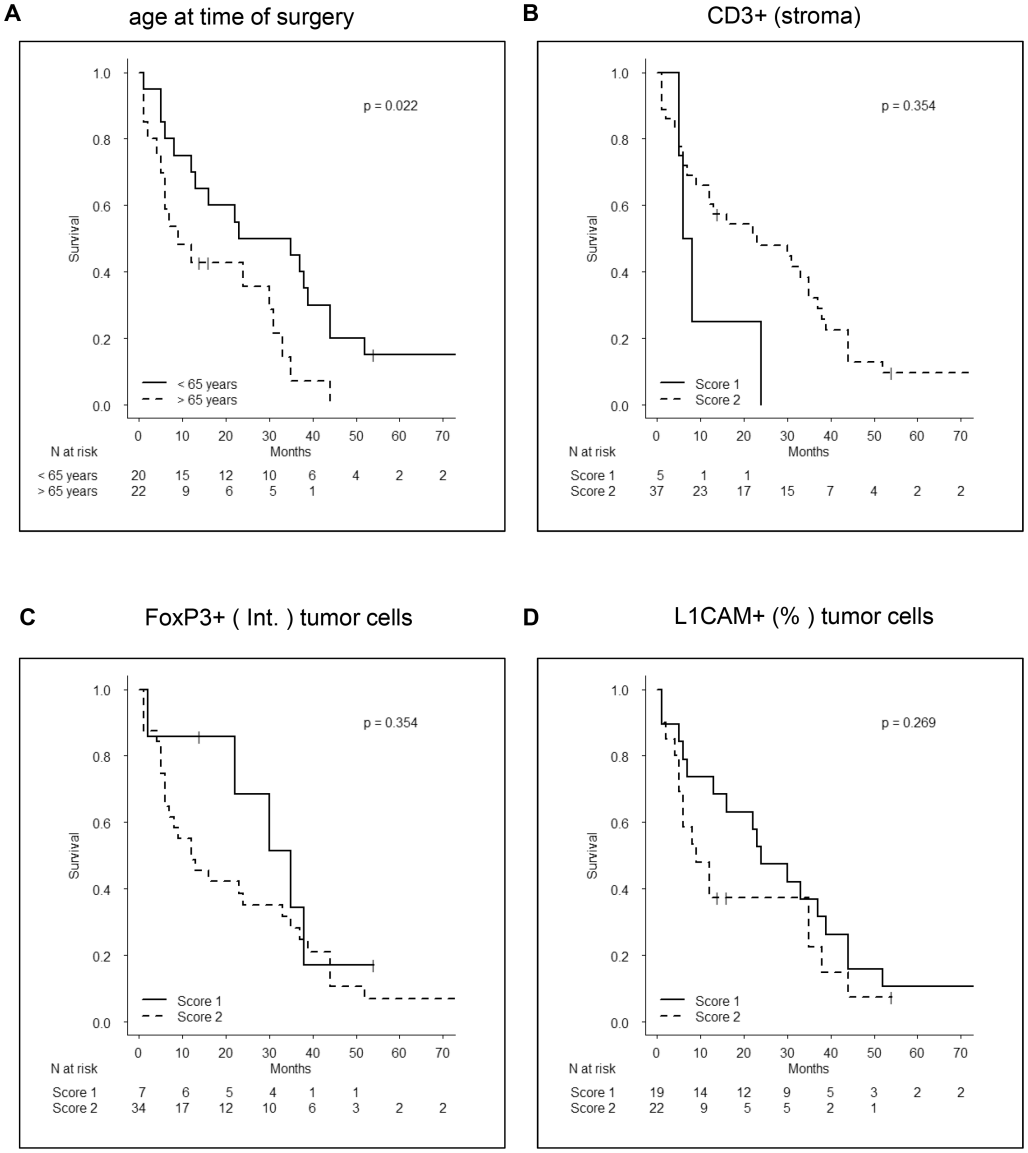
Correlation of stromal and carcinoma-related parameters with patient survival. Kapplan-Meyer-Curves show the impact of (**A**) age at time of surgery, (**B**) CD3+ cells/stroma, (**C**) FoxP3 expression in tumor cells (intensity) and (**D**) L1CAM expression in tumor cells (% cells) on survival of patients with T3N1M0 stage. Numbers at risk are depicted below each graph.

Finally, we examined the relationship between stroma and carcinoma-related parameters and patients survival. In contrast to previous reports, this study intended to evaluate the correlation of these parameters with survival of patients with the most frequently operated disease stage, namely tumor stage T3, nodal status N1 and no distant metastasis (M0). Only a higher age at time of diagnosis/surgery could be significantly correlated with shorter survival times (**Figure 6A**). Furthermore, higher numbers of CD3+ T cells (**Figure 6B**) as well as low expression of FoxP3 (**Figure 6C**) and L1CAM (**Figure 6D**) in carcinoma cells tended to be correlated with prolonged patient survival.

## Discussion

Both, CP and PDAC are characterized by an extensive and unique stromal reaction, which apparently promotes tumor development in the former and progression of the latter [4], [5], [12], [13], [27]. Profound experimental evidence *in vitro* and *in vivo* underscores the role of stromal cells in the initiation and progression of PDAC [22], [28]–[30]. Using an endogenous PDAC mouse model, Rhim et al. showed that inflammation can induce the epithelial-mesenchymal-transition (EMT) of pancreatic ductal epithelial cells thereby promoting cell invasion and dissemination already prior to the formation of primary tumors [31]. In accordance, our own studies demonstrated a role of myofibroblasts in the upregulation of L1CAM (as part of the EMT) not only in PDAC but already in non-transformed pancreatic duct (HPDE) cells, conferring an invasive and apoptosis resistant phenotype [16], [19], [22]. Furthermore, a role for macrophages in the induction of EMT in pancreatic tumor cells has been demonstrated [32], [33]. Likewise to FoxP3 [23], tumor cell associated expression of L1CAM has been shown to confer immunosuppressive functions to CD4+ T cells *in vitro* (unpublished observations). Based on these data, this study aimed at a comprehensive analysis of the correlation between stromal cells (T cells, macrophages, myofibroblasts) and ductal/carcinoma cell marker (proliferation, FoxP3, L1CAM and vimentin expression) in CP and PDAC as well as the prognostic significance of these variables in order to better integrate experimental data obtained by *in vitro* and *in vivo* studies.

Large numbers of stromal cells such as regulatory T cells, macrophages and myofibroblasts have been already correlated with short survival of PDAC patients [7], [8], [10], [12], [13]. However, all of these studies were conducted with a heterogeneous patient cohort e.g. including T1 to T4 tumor stages. In contrast, our study intended to test the prognostic significance of different stromal and tumor associated variables in PDAC patients with a defined, most frequently operated tumor stage T3N1M0. Only age below 65 years at time of surgery was significantly correlated with longer patient survival times. In addition, high levels of CD3 but low levels of FoxP3 and L1CAM in tumor cells tended to correlate with improved patient survival, being in line with previous reports demonstrating high tumor-associated expression of FoxP3 and L1CAM as negative prognostic marker [21], [24]. In summary, this study suggests that none of the determined variables is a suitable prognostic marker in patients with this advanced disease stage. To substantiate this finding, further analyses of larger patient cohorts are needed. One might further speculate whether the stromal composition and the markers detected in this study might be more suitable for the prediction of therapeutic responses as it has been shown for breast cancer patients [34].

A gene expression analysis already revealed that the stromal compartment in CP markedly differ from those in PDAC [35]. Overall, the present study discovered clear differences in the stromal composition in pancreatic tissues of CP and PDAC patients, too, albeit other markers were analyzed. Moreover, our study compared stromal and epithelial/tumoral parameters in CP and PDAC tissues considering the differentiation status (grading) of the tumors. Although our study included only a defined number of cases, this analysis clearly suggests that well differentiated tumors still have several features in common with CP tissues, whereas moderately and poorly differentiated tumors significantly differ from CP tissues. While CP tissues exhibited higher numbers of CD4+ T cells and macrophages, PDAC tissues were characterized by a higher abundance of regulatory T cells, and by trend, of myofibroblasts. In detail, greater numbers of CD4+, CD8+ and stromal γδ-T cells but smaller numbers of regulatory T cells and myofibroblasts were present in CP compared to PDAC.

Parallel staining of CD3 and CD8 in the consecutive sections indicated that γδ T cells were located in the same area of the stroma or the ductal epithelium as CD3- and CD8-positive cells in CP tissues. The different stromal localization of γδ-T cells in PDAC compared to CP patients might be explained by changes of stromal composition during progression to an invasive PDAC (see above). Moreover, in contrast to PDAC patients, γδ-T cells accumulated in the lymphoid follicles of CP patients suggesting a recruitment of γδ-T cells to the site of inflammation, whereas γδ-T cells in PDAC patients were mobilized to the tumor site (data not shown). The presence of γδ-T cells in the stroma adjacent to the tumor cells and their localization within the malignant epithelium of PDAC patients demonstrate that γδ-T cells infiltrate into PDAC. Although γδ-T cells have a low frequency in the peripheral blood as well as in the examined tissues, γδ-T cells accumulated in the ductal epithelium of PDAC patients close to the tumor cells. This accumulation of γδ-T cells in PDAC underlines an important role of γδ-T cells in the immune response against PDAC. However, their activity is apparently suppressed by the pronounced immunosuppressive microenvironment in PDAC. Regulatory T cells being known for their immunosuppressive function [4], were accordingly more abundant in PDACs than in CP. Additionally, myofibroblasts also contributing to an immunosuppressive microenvironment by releasing e.g. high amounts of TGF-β1 [4] tended to be more abundant in PDAC than in CP tissues. Thus, an efficient strategy to enhance γδ-T cell activity and to probably overcome immunosuppression of the stromal composition might be the usage of enhancer of γδ-T cell cytotoxicity [36]. In addition, strategies to eliminate or suppress regulatory T cells and myofibroblasts might be beneficial.

In contrast to regulatory T cells and myofibroblasts, macrophages were more abundant in CP than PDAC irrespective of their phenotype being characterized by high expression of M1-related HLA-DR or M2-related CD163. Interestingly, CPs did not only significantly differ from PDAC tissues with regard to the number of macrophages - as observed for γδ-T cells. While being distributed throughout the stroma in CP, macrophages were predominantly located in close proximity to tumor cells in PDAC tissues. Moreover, this tumor-related localization was associated with an increased vimentin expression in tumor cells, a higher abundance of myofibroblasts and a higher grading. Accumulation of myofibroblasts in turn correlated with increased L1CAM expression in ductal and tumor cells supporting experimental data on the role of macrophages and myofibroblasts in the induction of EMT by upregulating vimentin and L1CAM in ductal and PDAC cells [16], [33], [37]. Recently it has been shown, that not only macrophages with an anti-inflammatory phenotype (e.g. characterized by expression of CD163), which are supposed to exert pro-tumorigenic activities, promote EMT in premalignant and malignant pancreatic ductal epithelial cells but also pro-inflammatory macrophages (e.g. characterized by high HLA-DR expression) [33]. Taken together these findings from our group, the above mentioned findings from Rhim et al. [31] and the data from this study, there is strong evidence that macrophages play a central role in the induction of EMT and dedifferentiation of pancreatic ductal epithelial cells. Given the fact that macrophages are highly abundant in CP tissues still exhibiting more pro-inflammatory properties, EMT induction might be favoured by pro-inflammatory mediators already in CP and/or PanIN lesions. Accordingly, L1CAM as well as vimentin were considerably expressed already in ductal epithelial cells in CP tissues underscoring experimental data showing that inflammation promotes EMT in premalignant/precursor cells [16], [31].

In contrast to L1CAM, the role of tumor-related FoxP3 expression in PDAC development is less known. This study demonstrates that FoxP3 was already expressed in the ductal epithelium in CP tissues albeit to a lesser extent than in PDAC. Similar to L1CAM, low FoxP3 expression was correlated with low numbers of CD3+ T cells and myofibroblasts in CP and accordingly, expression level of FoxP3 positively correlated with L1CAM expression in pancreatic ducts. Preliminary results indicate that upregulation of L1CAM is FoxP3 dependent (unpublished observations). This might also point to a role of FoxP3 in EMT which is supported by the finding that high FoxP3 expression was associated with higher grading in PDAC patients. In accordance with previous findings suggesting an immunosuppressive role of FoxP3 in PDAC cells [23], high FoxP3 expression in tumor cells was associated with a low number of CD4+ T cells but elevated numbers of regulatory T cells as detected by FoxP3+ T cells.

Taken together, this study has revealed clear quantitative and qualitative differences in the stromal composition as well as in the phenotype of ductal and tumor cells in CP and PDAC, respectively. Moreover, our study indicates that dedifferentiated but not well differentiated tumors clearly differ from CP tissues on the basis of the markers analyzed in this study. Thus, in order to elucidate valid markers for a reliable discrimination of CPs and particularly well-differentiated tumors, a screening of more cases would be reasonable. We are aware of the fact that the explanatory power of the study would be higher with a larger cohort, however several interrelationships between stroma and ductal/carcinoma cells were discovered which in light of already published experimental data *in vitro* and in preclinical studies [31] strongly support the view that the inflammatory stroma contributes to malignancy-associated alterations such as EMT already in premalignant/precursor cells during CP.

## Supporting Information

Table S1 (DOCX)

Table S2 (DOCX)

Table S3 (DOCX)
