# Supplementary material for: Comparative Characterization of Stroma Cells and Ductal Epithelium in Chronic Pancreatitis and Pancreatic Ductal Adenocarcinoma
Source: PLoS One. 2014 May 5;9(5):e94357. doi: 10.1371/journal.pone.0094357 (PMC4010424; doi:10.1371/journal.pone.0094357)
Supplement: Table S1 — (DOCX) [file pone.0094357.s001.docx]

**Table S1: Interrelationship between stromal and ductal /tumor cell-related parameters in pancreatic tissues of CP patients and patients with a well-differentiated PDAC (grading G1)**

| **Variable** | **Code** | **CP (n=15)** | | | | | **PDAC G1 (n=6)** | | | | | **p-value** |
| --- | --- | --- | --- | --- | --- | --- | --- | --- | --- | --- | --- | --- |
| **Stromal compartment (related to)** |  | **Number of cases (Score)** | | | | | **Number of cases (Score)** | | | | |  |
| CD3+ (stroma) | A | 0(1) | 15(2) |  |  |  | 0(1) | 6(2) |  |  |  | np |
| CD8+ (stroma) | B | 1(1) | 13(2) |  |  | 1(NE) | 1(1) | 5(2) |  |  |  | 0.521 |
| CD4+ (stroma) | C | 1(1) | 14(2) |  |  |  | 4(1) | 2(2) |  |  |  | **0.011** |
| CD25+ (CD4+) | E | 12(1) | 3(2) |  |  |  | 3(1) | 3(2) |  |  |  | 0.291 |
| FoxP3+ (CD4+) | F | 11(1) | 4(2) |  |  |  | 2(1) | 4(2) |  |  |  | 0.146 |
| γδ+ (stroma) | G1 | 3(1) | 12(2) |  |  |  | 4(1) | 2(2) |  |  |  | 0.120 |
| γδ+ (duct/tumor cells) | G2 | 5(1) | 10(2) |  |  |  | 4(1) | 2(2) |  |  |  | 0.331 |
| CD68+ (stroma) | H1 | 4(1) | 11(2) |  |  |  | 2(1) | 3(2) |  |  | 1(NE) | 0.613 |
| CD68+ localization | H2 | 0(E/T) | 7(S) | 8(E) |  |  | 1(E/T) | 1(S) | 3(E) |  | 1(NE) | 0.157 |
| CD163+ (stroma) | I1 | 4(1) | 11(2) |  |  |  | 2(1) | 3(2) |  |  | 1(NE) | 0.613 |
| CD163+ localization | I2 | 0(E/T) | 7(S) | 8(E) |  |  | 1(E/T) | 1(S) | 3(E) |  | 1(NE) | 0.157 |
| HLA-DR+ (stroma) | K1 | 2(1) | 13(2) |  |  |  | 1(1) | 4(2) |  |  | 1(NE) | 1.000 |
| HLA-DR+ localization | K2 | 2(E/T) | 4(S) | 7(E) | 2(ND) |  | 1(ET) | 1(S) | 2(E) | 1(ND) | 1(NE) | 0.955 |
| αSMA+ (stroma) | L1 | 10(1) | 3(2) |  |  | 2(NE) | 3(1) | 2(2) |  |  | 1(NE) | 1.000 |
| αSMA^high^+ (αSMA+) | L2 | 7(1) | 6(2) |  |  | 2(NE) | 2(1) | 3(2) |  |  | 1(NE) | 0.268 |
| **Epithelial/tumoral compartment** |  |  |  |  |  |  |  |  |  |  |  |  |
| Ki67+ (%) | N | 11(1) | 1(2) |  |  | 3(NE) | 3(1) | 2(2) |  |  | 1(NE) | 0.191 |
| FoxP3+ (%) | O1 | 11(1) | 4(2) |  |  |  | 3(1) | 3(2) |  |  |  | 0.354 |
| FoxP3+ (intensity) | O2 | 10(1) | 5(2 |  |  |  | 2(1) | 4(2) |  |  |  | 0.331 |
| L1CAM+ (%) | P1 | 5(1) | 10(2) |  |  |  | 2(1) | 4(2) |  |  |  | 1.000 |
| L1CAM+ (intensity) | P2 | 13(1) | 2(2) |  |  |  | 2(1) | 4(2) |  |  |  | **0.031** |
| Vimentin+ (%) | R | 7(1) | 8(2) |  |  |  | 3(1) | 3(2) |  |  |  | 1.000 |

**ND**= not detectable; **NE**= not evaluable; **np** = not possible

**E/T**= located close to **E**pithelial/**T**umor cells; **S**= located in **S**troma; **E**= equally distributed close to epithelial/tumor cells and in stroma
